# Genome Sequence of the Acetogenic Bacterium *Oxobacter pfennigii* DSM 3222^T^

**DOI:** 10.1128/genomeA.01408-15

**Published:** 2015-12-03

**Authors:** Frank R. Bengelsdorf, Anja Poehlein, Bettina Schiel-Bengelsdorf, Rolf Daniel, Peter Dürre

**Affiliations:** a Institut für Mikrobiologie und Biotechnologie, Universität Ulm, Ulm, Germany; b Genomic and Applied Microbiology & Göttingen Genomics Laboratory, Georg-August University Göttingen, Göttingen, Germany

## Abstract

Here, we report the draft genome sequence of *Oxobacter pfennigii* DSM 3222^T^, an anaerobic, acetogenic, carbon monoxide-oxidizing, and butyrate-producing bacterium. The genome consists of a chromosome with a size of 4.49 Mbp.

## GENOME ANNOUNCEMENT

*Oxobacter pfennigii* DSM 3222^T^ (originally named *Clostridium pfennigii*) is an anaerobic endospore-forming bacterium, which was isolated from rumen fluid of a steer and described by Krumholz and Bryant in 1985 (1). In 1994, Collins et al. (2) compared phenotypic and phylogenetic data of clostridial species and found that *C. pfennigii* clearly merits a new genus. The closest autotrophic acetogenic relatives are, for instance, *Clostridium ljungdahlii* and *Clostridium carboxidivorans* (3). *O. pfennigii* does not use sugars or amino acids as an energy source, but catabolizes methoxylated aromatic compounds (e.g., vanilline, ferulate, caffeate, syringate) to butyrate (1). Moreover, it can reduce carbon monoxide and produce acetate and butyrate (1). As of yet, *O. pfennigii* is still the only member of its genus and not even a closely related bacterium has been described.

Chromosomal DNA of *O. pfennigii* was isolated using the MasterPure complete DNA purification kit (Epicentre, Madison, WI, USA). Illumina shotgun libraries were generated from the extracted DNA according to the protocol of the manufacturer. Sequencing was performed by employing a MiSeq system using MiSeq ReagentKit v3 (600 cycles), as recommended by the manufacturer (Illumina, San Diego, CA, USA), resulting in 4,476,360 paired end reads (300 bp) that were trimmed using Trimmomatic 0.32 (4).

The *de novo* assembly performed with the SPAdes genome assembler software 3.5.0 (5) resulted in 69 contigs (>500 bp) and an average coverage of 192-fold. The genome of *O. pfennigii* probably comprises a circular chromosome (4,510,552 bp) that has an overall G+C content of 39.0%. The software tool Prodigal (6) was used for automatic gene prediction, genes coding for rRNA and tRNA were identified using RNAmmer (7) and tRNAscan (8), respectively. The Integrated Microbial Genomes-Expert Review (IMG-ER) system (9) was used for automatic annotation, which was subsequently manually curated by using the Swiss-Prot, TrEMBL, and InterPro databases (10). The genome contains 9 rRNA genes, 66 tRNA genes, 3,328 protein-coding genes with predicted functions, and 949 genes coding for hypothetical proteins. The genome of *O. pfennigii* harbors genes encoding all proteins of the glycolysis pathway even though the bacterium does not use sugars as an energy source. Since no PTS (phosphoenolpyruvate [PEP]:carbohydrate phosphotransferase system) for sugars is found in the genome, the respective gene products of the glycolysis pathway probably serve only for gluconeogenesis.

With respect to the autotrophic acetogenic nature of *O. pfennigii*, we found genes encoding proteins of the methyl- and carbonyl-branches of the Wood-Ljungdahl pathway as well as of the Rnf (*Rhodobacter* nitrogen fixation) complex. The genes of the Wood-Ljungdahl pathway are not strictly clustered as shown for members of the genus *Clostridium* (11). Regarding the butyrate metabolism of *O. pfennigii*, genes encoding thiolase, 3-hydroxybutyryl-CoA dehydrogenase, crotonase, butyryl-CoA dehydrogenase, and electron-transfer-flavoproteins (A and B) are located in one cluster as in *Clostridium kluyveri* (12). Additionally, phosphotransbutyrylase- and butyrate kinase-encoding genes are clustered separately. Moreover, genes encoding proteins for nitrogenase, sporulation, and a glycine reductase were found in the genome of *O. pfennigii*.

### Nucleotide sequence accession numbers.

This whole-genome shotgun project has been deposited at DDBJ/EMBL/GenBank under the accession no. LKET00000000. The version described in this paper is version LKET01000000.
